# Pituitary-Targeted Knockout of Glucocorticoid Receptors Disrupts Growth Hormone Expression During Embryonic Development

**DOI:** 10.1210/endocr/bqaf119

**Published:** 2025-07-14

**Authors:** Scott Thomas Klug, Laura E Ellestad, Tom E Porter

**Affiliations:** Department of Animal and Avian Sciences, University of Maryland, College Park, MD 20742, USA; Department of Animal and Avian Sciences, University of Maryland, College Park, MD 20742, USA; Department of Poultry Science, University of Georgia, Athens, GA 30602, USA; Department of Animal and Avian Sciences, University of Maryland, College Park, MD 20742, USA

**Keywords:** somatotroph, glucocorticoid receptor, pituitary, development, growth hormone

## Abstract

Numerous studies have implicated glucocorticoids in the regulation of somatotroph differentiation. However, few studies have investigated a requirement for glucocorticoid receptors (GR) in this process. We hypothesized that GR is essential for the normal ontogeny of pituitary growth hormone (GH) during mouse embryonic development. Anterior pituitary cells were isolated from e12.5 to e13.5 mice and e11 chickens and cultured with or without corticosterone (CORT) in the absence or presence of ZK98299, a GR-specific antagonist. CORT induced *GH* mRNA expression in pituitary cells from both species, and this response was blocked by inclusion of the GR antagonist. Mouse embryos with pituitary-targeted knockout of GR were generated utilizing the cre-LoxP Recombinase system under control of the pituitary-specific alpha-glycoprotein subunit (*αGSU*) promoter. All cre-positive *GR*^(−/−)^ embryos died at birth. Therefore, anterior pituitary, brain, heart, liver, and muscle tissues, were collected on embryonic days 17.5/18.5 for RNA isolation and RT-qPCR analysis. *Cre* mRNA expression was only found in the pituitary, and *GR* mRNA levels were significantly decreased in the pituitaries of *GR*^(−/−)^ embryos. *GH* mRNA was significantly decreased in pituitary-targeted *GR*^(−/−)^ knockout embryos in comparison to wild-type *GR*^(+/+)^ embryos. Significant differences in expression of other pituitary hormones in *GR*^(−/−)^ embryos were not observed, indicating that the effect of pituitary-targeted knockout of *GR* was restricted to disruption of *GH* gene expression. To our knowledge, this is the first report that homozygous *GR* knockout in the anterior pituitary gland in mice suppresses embryonic *GH* expression, confirming an essential role for *GR* signaling in the normal ontogeny of somatotrophs.

The anterior pituitary gland consists of 5 unique endocrine cell types, including somatotrophs, lactotrophs, gonadotrophs, thyrotrophs, and corticotrophs, which release growth hormone (GH), prolactin (PRL), luteinizing hormone (LH) and follicle-stimulating hormone (FSH), thyroid-stimulating hormone (TSH), and adrenocorticotrophic hormone (ACTH), respectively. The first endocrine cell type to differentiate is the corticotrophs (1). The production of ACTH from corticotrophs stimulates the production of glucocorticoids (GCs), specifically corticosterone (CORT) in birds and rodents, from the adrenal glands during development. CORT binds to the glucocorticoid receptor (GR), which acts as a ligand-activated transcription factor and regulates numerous genes (2). CORT has been shown in vitro and in vivo to prematurely differentiate chicken and rat somatotrophs and to induce *GH* mRNA expression (3-19). Based on these previous findings, we hypothesized that GR is essential for the normal ontogeny of *GH* gene expression. Previous studies have investigated the role of GCs in somatotroph differentiation and *GH* mRNA expression; however, to our knowledge, none have specifically investigated the effect of GR knockout in the pituitary. This may be due to the fact that whole-body knockout of *GR* in mice leads to neonatal death as a result of immature lungs (20). In order to investigate the role of GR in somatotroph differentiation and *GH* mRNA expression in mice, we generated homozygous GR pituitary knockout mouse embryos utilizing the cre-LoxP recombinase system under control of the pituitary-specific common alpha-glycoprotein subunit (*αGSU*) promoter. The *αGSU* gene is expressed throughout the pituitary gland prior to differentiation of any of the hormone-producing cell types (1, 21), likely in the progenitor cell for all pituitary endocrine cell types. Thus, GR should be knocked out in the somatotroph precursor cells in this transgenic mouse model, allowing us to determine the requirement for GR in somatotroph differentiation.

## Methods

All studies were approved by the Institutional Animal Care and Use Committee at the University of Maryland. Wild-type C57 Black/6 mice were purchased from Charles River Laboratories or Jackson Laboratories. Fertile wild-type Ross chicken eggs were obtained from Perdue Farms (Salisbury, MD).

### Anterior Pituitary Cell Dispersions

Wild-type C57 Black/6 mice breeding pairs were established, with male mice removed after 2 days to produce timed-pregnant females. The morning after introduction of males was defined as embryonic day (e)0.5. Pregnant mice were euthanized 14 days after introduction of the male in the breeding pair, and mouse embryo pituitary glands were collected as described below. Similarly, anterior pituitary glands were isolated from embryonic day (e)11 chicken embryos and dissociated into single cells as follows. Dissected anterior pituitary glands from e12.5/e13.5 mouse embryos and e11 chicken embryos were placed into separate sterile petri dishes containing minimum essential medium (MEM; Sigma-Aldrich) and cleaned of posterior pituitary and connective tissue. For each replicate experiment, pituitary glands were pooled from 8 to 16 mouse embryos and 10 to 20 chicken embryos. Anterior pituitaries were dissociated into single cells as previously described (22). Briefly, anterior pituitary glands were removed from the petri dishes and placed in 10 mL of minimum essential medium containing trypsin (1 mg/mL; Difco, Detroit, MI), and incubated (37 °C, 95% O_2_ and 5% CO_2_) for 45 minutes. A flame-polished, siliconized Pasteur pipette was used to gently triturate the pituitaries at 15-minute intervals. After the pituitary tissues were dispersed, they were washed twice with 10 mL of Dulbecco's modified Eagle's medium (DMEM; ThermoFisher) and centrifuged. The trypan blue dye-exclusion method was conducted to determine viability of the cells, which was consistently greater than 90% for all groups.

### Cell Culture

All chemicals were obtained from Sigma Chemical Company, unless otherwise stated. Cell culture media and reagents were obtained from Invitrogen (Carlsbad, CA). Dispersed chicken and mouse pituitary cells were incubated for 16 hours (37 °C, 5% CO_2_) in poly-L-lysine-coated cell culture plates in DMEM, supplemented with 0.1% bovine serum albumen (BSA), 100 U/mL of penicillin G, 100 µg/mL of streptomycin sulfate, and 5 µg/mL of human insulin, with or without any treatments. To determine the role GR plays in regulating embryonic *GH* mRNA expression in response to corticosterone (CORT), both chicken (2 × 10^5^/well) and mouse (1 × 10^5^/well) pituitary cells were divided into 2 groups, one with a GR antagonist (ZK98299, Schering, AG, 10^−9^ M final concentration), and one with vehicle (media). Cells from each group were collected 16 hours after the addition of CORT (10^−9^ M final concentration) for total RNA extraction (RNeasy Mini Kits, Qiagen) and analysis by reverse transcription–quantitative polymerase chain reaction (RT-qPCR). Three independent replicate experiments were performed with mouse embryonic pituitary cells, and 2 independent replicate experiments were performed with chicken embryonic pituitary cells.

### Pituitary-Targeted *GR* Knockout Mice

C57 Black/6 mice with pituitary-targeted glucocorticoid receptor (*GR*; *Nr3c1*) heterozygous knockouts were created by breeding 2 separate transgenic mouse lines, utilizing the cre-LoxP Recombinase system. The first transgenic mouse line contained a cre cassette inserted downstream from 4.6 kb of the mouse *αGSU* promoter (*Cga*). *αGSU* encodes for the common α-subunit of the 3 glycoprotein hormones that are synthesized and secreted by the anterior pituitary, including follicle-stimulating hormone (FSH), luteinizing hormone (LH), and thyroid-stimulating hormone (TSH). *αGSU* mRNA is easily detected in thyrotrophs and gonadotrophs of mice by e11.5, making it the earliest marker of differentiation of a hormone-producing cell in the pituitary; however, it is expressed throughout the entire pituitary gland during earlier development (21). In this model, the *cre* recombinase gene is expressed in cells when the *αGSU* gene is expressed. The *αGSU*-cre mice were generated as previously described (23) and were purchased from Jackson Laboratories (JAX stock #004426). The second transgenic mouse line contained LoxP sites flanking exon 2 of *Nr3c1* (*GR*) and were generated as previously described (24). Exon 2 of *Nr3c1* contains the translational start site for *GR*. These *Nr3c1*-floxed mice were generously provided by Dr. Louis J. Muglia (Washington University School of Medicine, Saint Louis, Missouri).

The *αGSU*-cre mice were bred with *Nr3c1*-floxed mice to establish offspring that were heterozygous positive for the floxed-glucocorticoid receptor (*GR*) and positive for *cre*, designated *GR*^(+/−)^. *GR*^(+/−)^ mice were outcrossed with wild-type C57 Black/6 mice for at least 6 generations to establish 6 unrelated paternal families of *GR*^(+/−)^ mice for experimental use. Females were bred first to maintain the colony of 6 unrelated paternal families. Then, breeding pairs of *GR*^(+/−)^ mice (ages 7 to 11 months) were established after genotyping confirmed all mice were *GR*^(+/−)^ and *cre*-positive. Initially, females were allowed to give birth to their litters. However, we determined that zero homozygous floxed-*GR*, *cre*-positive pups, designated as *GR*^(−/−)^, survived. To overcome the lethality of the *cre*-positive/*GR*^(−/−)^ genotype, timed-pregnant females were generated. To do so, male mice were removed from breeding cages 2 days after their introduction to the females, and the dams were sacrificed 19 days after introduction of the males. This method created an accurate fertilization timing window between these 2 days, confidently producing mouse pups at e17.5 or e18.5 of gestation, after the age at which somatotroph populations should be established.

### Tissue Dissection for mRNA Quantification

All dissections were performed in a laminar-flow hood, and dissection tools were sterilized prior to each experiment. Pregnant mice were sacrificed at e17.5/e18.5 by CO_2_ asphyxiation. Forceps and dissecting scissors were used to make an opening in the ventral body cavity of the pregnant mice. The embryos were removed from the uterus and rapidly decapitated. The head from each embryo was placed on sterile gauze under a dissecting microscope. The cranium of each embryo was opened using sterile scissors, and the pituitary gland was removed and placed in a petri dish containing sterile minimum essential medium. Anterior pituitary glands were separated from the posterior pituitary and connecting tissue and were immediately placed into liquid nitrogen. Whole brain tissue was also collected. As the anterior pituitary gland and whole brain tissue were being collected, dissecting scissors were used to make an opening in the ventral body cavity of the embryos, exposing the chest and abdominal cavities. Forceps and dissecting scissors were used to collect liver, heart, and muscle tissue (from the left hind-leg). Tail snips were also collected from each embryo for DNA extraction and genotyping, as described below. Forceps and dissecting scissors were washed and sterilized with 70% ethanol between each mouse embryo dissection. Breeding pairs were established within each of the paternal families, and those litters subsequently confirmed by PCR genotyping to contain a *GR*^(−/−)^ pup were analyzed further (*n* = 3 separate paternal families).

### DNA Extraction

Genomic DNA was extracted from mouse tail snips using the QIAamp Fast DNA Tissue kit (Qiagen, Cat: 51404), according to manufacturer's instructions. Briefly, tail snips in microcentrifuge tubes were cut into smaller pieces using a clean scalpel blade. The following kit reagents were added at the following volumes to each microcentrifuge tube: 200 µL AVE, 40 µL VXL, 1 µL DX reagent, 20 µL Proteinase K, and 4 µL RNase A (100 mg/mL). Microcentrifuge tubes were shaken (270 rpm) in an incubator (37 ^o^C) overnight to dissolve all tissue. The following day, 265 µL Buffer MVL were added to each tube and vortexed. The mixture from each microcentrifuge tube was transferred to a QIAamp mini spin column in a clean 2 mL collection tube. Collection tubes were centrifuged, and each spin column was placed into a new collection tube (all centrifuge steps were performed for 30 seconds at maximum speed unless stated otherwise). Buffer AW1 (500 µL) was added to each spin column, and all collection tubes were centrifuged. Spin columns were then placed into new collection tubes. Buffer AW2 (500 µL) was added to each spin column, and all collection tubes were centrifuged. All spin columns were then transferred to new collection tubes and centrifuged for 2 minutes at maximum speed to remove any residual buffer from the spin columns. After centrifugation, all spin columns were placed into sterile 1.5 mL microcentrifuge tubes. DNA elution buffer (50 µL) was carefully pipetted directly onto the filter of each spin column. After 1 minute of incubation at room temperature, all microcentrifuge tubes were centrifuged for 1 minute to collect the purified DNA.

### Polymerase Chain Reaction Genotyping

Polymerase chain reaction (PCR) reactions (20 µL) were conducted to genotype each mouse. Table 1 provides sequences for all primers used to genotype mice. Working solutions containing the forward and reverse primers (10 µM) were made for mouse *gr* (WT), flanking loxp sequences (FLOX), and *cre* (CRE). Mouse *gh* (GH) primers were used as an internal positive control for each PCR reaction. Samples previously determined to be negative for either *gr*, loxp sequences, or *cre*, were used as negative controls to provide confidence that no contamination was present in the reactions. Optimized PCR reaction recipes were the same for the FLOX primers and the WT primers. For individual sample reactions, 1 µL of template DNA (30 to 50 ng/µL) was added to a well containing 0.3 µL of GH primers, 1 µL of WT or FLOX primers, and 17.7 µL of GoTaq (Promega). When samples were genotyped for CRE, 1 µL of template DNA (30 to 50 ng/µL) was added to a well containing 0.3 µL CRE primers, 1 µL GH primers, and 17.7 µL GoTaq. A thermocycler was set with the following cycling program: a 5-minute step at 95 °C, followed by 35 cycles comprising of 95 °C for 45 seconds, 57 °C for 30 seconds, and 72 °C for 1 minute. After 35 cycles, a final 72 °C extension step occurred for 7 minutes before being lowered to 4 °C.

**Table 1.** Primers used for PCR to genotype mouse DNA

Gene	Forward primer (5′ to 3′)	Reverse primer (5′ to 3′)
GH	CCCTCATCCTAGTGAACAAACA	AGTTGGAACGCACTCACATTA
GR (WT)	GGCATGTTAGAAACTGGAAAGGA	CAGTTCTTAACCCTCTCATTGAAAGGT
GR (FLOX)	GGCATGTTAGAAACTGGAAAGGA	CAATAGCAGGCAACAACTTCGT
CRE	CGATGCTTTTAAACCTGTGAGAGTT	CACGTAACAGACGTTTTCAGATACCT

### Gel Electrophoresis

Once the PCR reactions were completed, 20 µL of 1× loading buffer (Biorad) were added to each well. A clean, rubber seal was placed on individual PCR plates to allow the plates to be vortexed. After reactions were mixed, the entire plate was centrifuged, and 8 µL from each well were loaded into a 1% agarose gel for electrophoresis. Duplicate reactions for each sample and primer combination were run in adjacent wells. Agarose (2.5 g, Genesee, Cat:20-102) was dissolved in a flask containing 250 mL of TE buffer and 7.5 µL of ethidium bromide (10 mg/mL, Biorad, Cat:20-102). Once the gel solidified, PCR plates were loaded into wells with a DNA ladder at the end of each lane. Gels were run at 130 V for 60 to 90 minutes.

### Total RNA Extraction

Total RNA was extracted using a RNeasy Mini Kit (Qiagen, Cat: 74106) according to the manufacturer's instructions. Mouse tissue samples were removed from a −80 °C freezer and placed into liquid nitrogen. The homogenizer (Scilogex) was cleaned before the first sample, in between each sample, and after the last sample was homogenized. Buffer RLT (600 µL) was added to each sample. Samples were then homogenized for approximately 30 seconds, gently mixing the cryotube up and down. On-column DNase digestion was used to reduce levels of contaminating DNA. Samples were eluted in 60 µL of RNase-free water and quantified using the Quant-it RiboGreen assay kit (Invitrogen).

### Analysis of mRNA Levels by RT-qPCR

Two-step RT-qPCR was performed to quantify mRNA levels in each embryonic tissue across genotypes. Each reverse transcription reaction (20 µL) consisted of 1 µL of 50 µM oligo-dT primer, 1 µL of 10 mM dNTPs, 4 µL of 5× first strand buffer, 1 µL of 0.1 M DTT, 1 µL of an RNase inhibitor (10 U/µL), 1 µL of SuperScript III (200 U/µL) and 1 µg of total RNA, except for the mouse pituitary samples due to low RNA recovery (50 to 200 ng). Negative controls for genomic DNA contamination were created by pooling RNA from each tissue and processing them in the same way as the other samples, but without SuperScript III (no RT control). All reactions were diluted with 180 µL of RNase-free water, except the mouse pituitary reactions, which were diluted with 20 µL of RNase-free water. Each PCR reaction (15 µL) consisted of 0.6 µL forward primer, 0.6 µL reverse primer, 5.3 µL of autoclaved distilled water, 7.5 µL of 2× QuantiTect SYBR Green PCR master mix (Qiagen), and 1 µL of cDNA.

Annealing temperatures for each primer varied slightly; however, the remaining thermal cycling parameters were held consistent and are as follows: 1 cycle of 95 °C for 15 minutes, followed by 40 cycles of 95 °C for 10 seconds, 54 or 55 °C for 30 seconds, and 75 °C for 30 seconds. Finally, a melt curve analysis (held at 65 °C for 5 seconds and then increased to 95 °C in 0.5 °C increments) was performed. Primer sequences of each gene used in RT-qPCR are listed in Table 2. Levels of *β-actin* (*ACTB*) mRNA were quantified in every sample. Statistical analysis indicated that levels of *ACTB* mRNA differed by tissue (*P* < .05) but did not differ by genotype within any individual tissue. These results confirmed that *β-actin* was a suitable gene for normalization of individual mRNA levels within each tissue. After normalization, all data were analyzed using the 2^−ΔΔCt^ equation, where Ct equates to the cycle number when the amount of amplified cDNA product reached a fixed threshold of fluorescence for each sample. Data for *cre* and *GR* mRNA expression are presented across all tissues by genotype. Data for mRNA levels for pituitary hormones are presented in the pituitary samples by genotype.

**Table 2.** Primers used for reverse transcription–quantitative PCR

Gene	Forward primer (5′ to 3′)	Reverse primer (5′ to 3′)
Mouse		
β-actin	GATTACTGCTCTGGCTCCTAGCAC	GACAGTGAGGCCAGGATGGA
GH	TTCTAATGCTGTGCTCCGAGC	AATGGAATAGCGCTGTCCCTC
TSHβ	GGAGAGAGTGTGCCTACTGCCT	CCTGAGAGAGTGCATATTTGGGA
PRL	AGAAGCCCCCGAATACATCC	TCCCATTTCCTTTGGCTTCA
POMC	CCATAGATGTGTGGAGCTGGTG	TCCAGCGAGAGGTCGAGTTT
LHβ	CCCAGTCTGCATCACCTTCA	TAGGTGCACACTGGCTGAGG
FSHβ	ACCAGCTTTCCCTCACATGC	CAGGTGTGTTTGTAGGCAAGCTAA
α-GSU	TTCCAAAGAATATTACCTCGGAGG	GCTACAGTGGCACTCCGTATGAT
Pit1	CAAACGAAAGGAAGAGGAAACG	AGCCATCCGCATGATCTCC
GR	GAGGACAACCTGACTTCCTTG	AACTCACATCTGGTCTCATTCC
CRE	GCTGGAGTTTCAATACCGGAGA	CATTGCCCCTGTTTCACTATCC
Chicken		
GH	CACCTCAGACAGAGTGTTTGAGAAA	CAGGTGGATGTCGAACTTATCGT

### Statistical Analyses

Results for *GH* mRNA levels in cultured pituitary cells were analyzed by two-way analysis of variance using the (Statistical Analysis System (SAS; Cary, NC) on results from 3 separate experiments for mouse embryonic pituitary cells and 2 separate experiments for chicken embryonic cells. Levels of mRNA were determined in tissues from those mouse litters subsequently confirmed to contain a *GR*^(−/−)^ pup (*n* = 3 separate paternal families). *Cre* and *GR* mRNA expression data are reported as the means ± SEM for each genotype for each tissue. Data were analyzed by one-way analysis of variance within tissue using the PROC ANOVA procedure in SAS. Tukey's post hoc analysis was conducted to determine statistical significance between genotypes for each tissue. *αGSU*, *β-actin*, *Pit1*, *GH*, *TSHβ*, *FSHβ*, *LHβ*, *PRL*, and *POMC* mRNA expression data from e17.5/e18.5 mouse embryos are reported as the means ± SEM for each genotype from pituitary tissue. Data were analyzed by one-way analysis of variance using the PROC ANOVA procedure in SAS. Tukey's post hoc analysis was conducted to determine statistical significance between genotypes for each gene.

## Results

### Effects of CORT and a *GR* Antagonist on *GH* mRNA In Vitro

**Figure 1.**
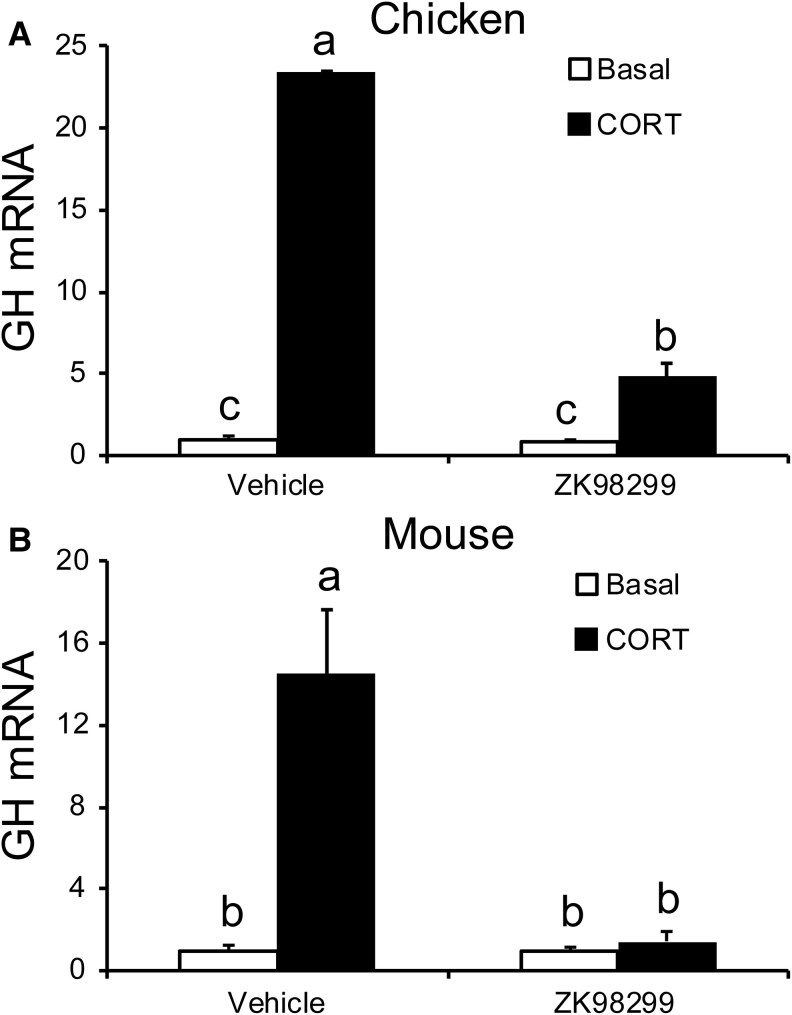
*Gh* mRNA expression in pituitary cells derived from e11 chicken and e12.5/e13.5 mice embryos. All results were normalized to *Gh* mRNA levels in cells cultured under basal conditions. Data are shown as means ± SE from 3 separate experiments with mouse embryonic pituitary cells and 2 separate experiments with chicken embryonic pituitary cells. A) *Gh* mRNA expression in e11 chicken somatotrophs in response to CORT, in the absence or presence of a glucocorticoid receptor antagonist (ZK98299). B) *Gh* mRNA expression in e12.5/e13.5 mouse somatotrophs in response to CORT, in the absence or presence of a glucocorticoid receptor antagonist. Within each species, significant differences are shown using different letters.

To provide a foundation of in vitro data for our in vivo experiments in mice, we isolated and cultured pituitary cells from e11 chicken and e12.5/e13.5 mouse wild-type embryos. To investigate the role *GR* plays in *GH* mRNA expression, we treated pituitary cells from each species with and without a *GR* antagonist. ZK98299, a *GR* antagonist in chickens and mice (25), was added to the media with or without CORT. *GH* mRNA levels were analyzed by RT-qPCR 16 hours after CORT and ZK98299 treatment. Results are presented in Fig. 1. Treating e11 chicken pituitary cells with CORT increased *GH* mRNA expression, relative to basal, in the cultures without ZK98299. This response to CORT was blocked when ZK98299 was present, indicating that the *GR* is required for CORT-induced *GH* mRNA expression in chicken embryonic pituitary cells. These results are consistent with previous in vitro and in vivo findings of CORT induction of *GH* mRNA expression in chicken embryonic pituitary cells (3, 9, 13, 14, 26). Similar results were observed with e12.5/e13.5 mouse pituitary cells. Again, an increase in *GH* mRNA expression was observed after CORT treatment, relative to basal, in the absence of ZK98299. The *GH* mRNA response to CORT treatment was blocked when ZK98299 was present in the media, comparable to the inhibition observed in the chicken pituitary cells. This experiment demonstrated that *GR* is required for CORT to increase *GH* mRNA expression in both mouse and chicken embryonic pituitary cells. In mice, *GH* mRNA first appears on e15.5 (21). However, in the present study, effects of CORT on *GH* mRNA expression were analyzed before normal GH ontogeny. Therefore, this study supports similar findings that glucocorticoid treatment can lead to premature differentiation of somatotrophs and the production of *GH* mRNA (3, 5, 6, 16, 27-29). Furthermore, it defines a requirement for *GR* in the GH response to CORT in vitro in both birds and mammals.

### Pituitary-Targeted Knockout of *GR* Is a Neonatal Lethal Phenotype in Mice

Having confirmed that CORT can prematurely induce *GH* mRNA in vitro and that this response is blocked by a GR antagonist, we next sought to evaluate the requirement for *GR* in normal GH expression in vivo using the pituitary-targeted *GR* knockout mice that we generated. However, all *GR*^(−/−)^ pups died at birth. Table 3 presents genotypes of pups born, and Table 4 presents genotypes for e17.5/e18.5 embryos from cre-positive, heterozygous *GR*^(+/−)^ breeding pairs. Table 3 presents genotyping data of pups from *GR*^(+/−)^ breeding pairs from 3 separate paternal family lines. DNA samples from pups were collected between 10 and 28 days of age. Pup genotyping data (*n* = 14) were analyzed as cre-positive or cre-negative for each genotype (*GR*^(+/+)^, *GR*^(+/−)^, *GR*^(−/−)^). Zero pups were genotyped as cre-negative-*GR*^(+/−)^, cre-negative-*GR*^(−/−)^, or cre-positive-*GR*^(−/−)^. 36% of the pups (*n* = 5) were cre-negative-*GR*^(+/+)^. 57% of the pups (*n* = 8) were cre-positive-*GR*^(+/−)^. Only 7% of the pups (*n* = 1) were cre-positive-*GR*^(+/+)^. When only cre-positive pups (*n* = 9) were analyzed by genotype, 0% of the pups were *GR*^(−/−)^, 11% of the pups were *GR*^(+/+)^ (*n* = 1), and 89% of the pups were *GR*^(+/−)^ (*n* = 8). These results indicate that no cre-positive-*GR*^(−/−)^ mice were present after birth.

**Table 3.** Table of genotypes by percent from *GR*^(+/−)^ breeding pairs

Paternal family	Total # of pups on day of birth	GR(+/−) ; CRE (−)	GR(+/+) ; CRE (−)	GR(−/−) ; CRE (−)	GR(+/−) ; CRE (+)	GR(+/+) ; CRE (+)	GR(−/−) ; CRE (+)
E	6	0	2	0	3	1	0
		0%	33%	0%	50%	17%	0%
C	4	0	2	0	2	0	0
		0%	50%	0%	50%	0%	0%
G	4	0	1	0	3	0	0
		0%	25%	0%	75%	0%	0%
	Average %	0%	36%	0%	57%	7%	0%
	% among CRE + pups				89%	11%	0%

Genotypes by percentage from pups born from separate paternal family lines.

**Table 4.** Table of genotypes by percent from *GR*^(+/−)^ breeding pairs

No. of litters	Total # of embryos on e18.5	GR(+/−) ; CRE (−)	GR(+/+) ; CRE (−)	GR(−/−) ; CRE (−)	GR(+/−) ; CRE (+)	GR(+/+) ; CRE (+)	GR(−/−) ; CRE (+)
3	18	0	4	0	6	5	3
	Average %	0%	22%	0%	33%	28%	17%
	% among CRE + pups				43%	36%	22%

Genotypes by percentage from embryos collected on e17.5/e18.5.

Table 4 presents genotyping data from e17.5/e18.5 embryos from cre-positive-*GR*^(+/−)^ breeding pairs from 3 paternal family lines. As both the dam and sire were cre-positive, the embryos could have 1 or 2 *cre* alleles and this could not be discerned by genotyping. Embryo genotyping data (*n* = 18) were analyzed as cre-positive or cre-negative for each genotype (*GR*^(+/+)^, *GR*^(+/−)^, *GR*^(−/−)^). Similar to data from Table 3, zero embryos were found to be cre-negative-*GR*^(+/−)^ or cre-negative-*GR*^(−/−)^. Among all embryos, 22% of the embryos (*n* = 4) were cre-negative-*GR*^(+/+)^, 33% of the embryos (*n* = 6) were cre-positive-*GR*^(+/−)^, and 28% of the embryos (*n* = 5) were found to be cre-positive-*GR*^(+/+)^. Interestingly, 17% of the embryos (*n* = 3) were found to be cre-positive-*GR*^(−/−)^. When only the cre-positive embryos (*n* = 14) were analyzed by genotype, 43% were *GR*^(+/−)^ (*n* = 6), 36% were *GR*^(+/+)^ (n = 5), and 22% were *GR*^(−/−)^ (*n* = 3). Thus, cre-positive-*GR*^(−/−)^ embryos survived to e19.5 but died around the time of birth.

### Effects of Pituitary-Targeted *GR* Knockout on *GH* mRNA In Vivo

**Figure 2.**
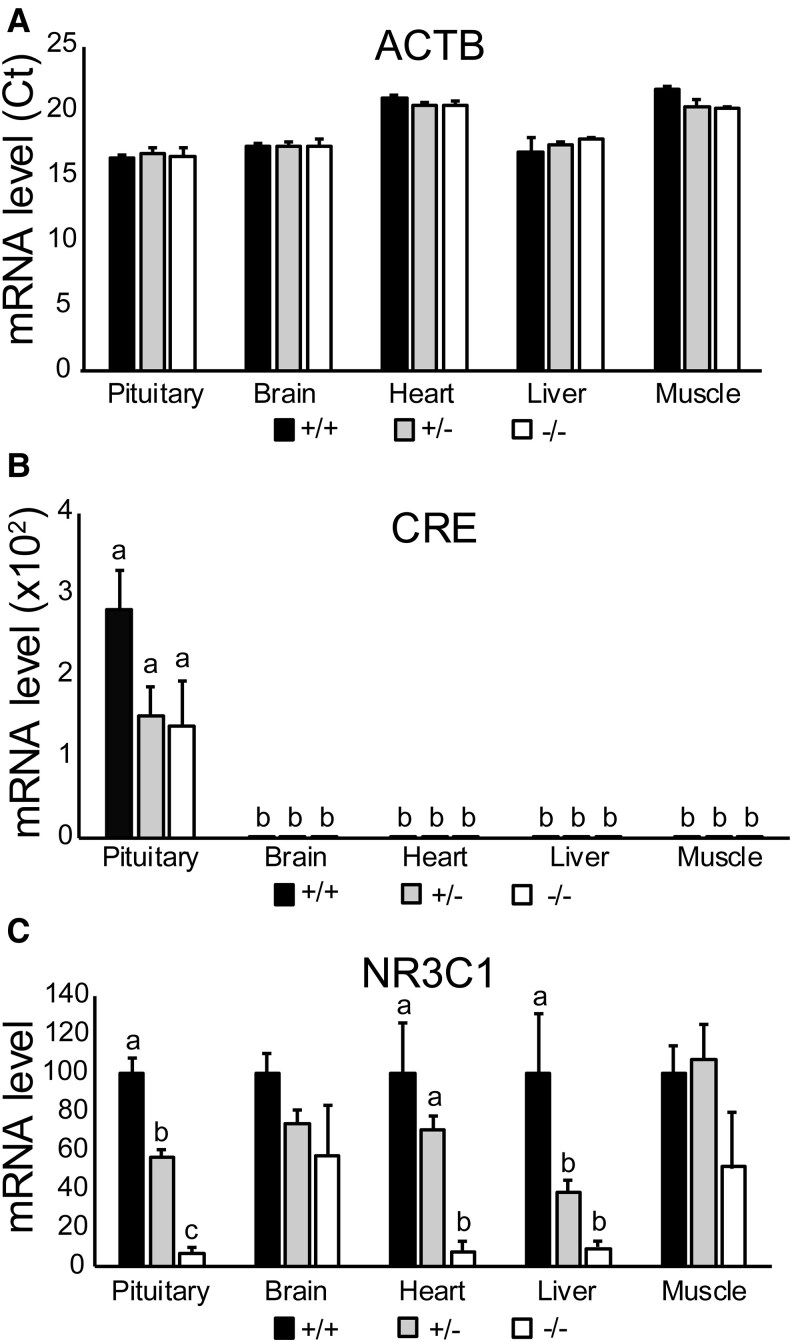
Relative *Actb*, *Cre*, and *Nr3c1* (*GR*) mRNA expression across tissues by genotype in e17.5/e18.5 mice. Pituitary, brain, heart, liver, and muscle tissues were dissected from e17.5/e18.5 mice (*n* = 11). DNA was collected from tail snips to genotype each embryo as *GR*^(+/+)^ (*n* = 3), *GR*^(+/−)^ (*n* = 5), or *GR*^(−/−)^ (*n* = 3). Total RNA was isolated from all tissues for RT-qPCR. Data are shown as means ± SE. One-way ANOVAs were conducted for each tissue, followed by Tukey's test for post hoc analysis. There were no statistical differences between genotypes within any tissue. (A) Relative *Actb* mRNA expression across tissues by genotype. (B) Relative *Cre* mRNA expression across tissues by genotype. (C) Relative *Nr3c1* exon 2 mRNA expression across tissues by genotype normalized to *Actb* mRNA levels. One-way ANOVAs were conducted for each tissue, followed by Tukey's test for post hoc analysis. Significant differences within tissues are shown using different letters. Compared to *GR*^(+/+)^ and *GR*^(+/−)^ embryos, *Nr3c1* mRNA expression in *GR*^(−/−)^ embryos was significantly reduced within the heart (*P* = .002) and liver (*P* = .004). In the pituitary, *Nr3c1* mRNA expression for each genotype was significantly different from the other genotypes (*P* = .0001), with levels for *GR*^(+/+)^ embryos being the greatest, *GR*^(+/−)^ embryos being intermediate, and *GR*^(−/−)^ embryos being the lowest. There were no significant differences in *Nr3c1* mRNA expression between any genotype for the brain (*P* = .11) and the muscle (*P* = .14).

As all cre-positive *GR*^(−/−)^ embryos survived to e18.5 but died around the time of birth, timed-pregnant litters were sacrificed on e17.5/e18.5 from 3 separate paternal families, and levels of mRNA were quantified. Figure 2A presents levels of *β-actin* mRNA. Figure 2B presents relative *cre* mRNA expression, normalized to *β-actin*, by tissue and genotype. A two-way ANOVA was conducted to compare *cre* mRNA expression across all tissues and genotypes. *Cre* mRNA expression was only detected in the anterior pituitary, and it was significantly greater than all other tissues (*P* = .0001). A one-way ANOVA was conducted on the pituitary data, followed by a Tukey's test for post hoc analysis. *Cre* mRNA expression was not statistically different among any genotypes within the pituitary tissue (*P* = .06). *Cre* mRNA expression in the wild-type (*GR*^(+/+)^) embryos was 0.028 (n = 3). *Cre* mRNA expression of the floxed (*GR*^(−/−)^) embryos (*n* = 3) was 0.013. *Cre* mRNA expression of heterozygous (*GR*^(+/−)^) embryos (*n* = 5) was 0.015. However, these apparent differences were not statistically different. These results indicate that cre expression was restricted to the anterior pituitary gland and did not differ within the anterior pituitary gland among genotypes on e17.5/e18.5.

Figure 2C presents relative *GR* mRNA expression, normalized to *β-actin* and wild-type mice, by tissue and genotype. One-way ANOVAs were conducted for each tissue, followed by Tukey's tests for post hoc analysis. Significant differences between genotypes for each tissue are shown using different letters. There were no significant differences in *GR* mRNA expression between any genotype for the brain (*P* = .11) and the muscle (*P* = .14). *GR* mRNA expression in the heart of *GR*^(−/−)^ mouse embryos (*n* = 3) was significantly less (*P* = .002) compared to *GR*^(+/−)^ mice (*n* = 5) and *GR*^(+/+)^ mice (*n* = 3). In the liver, *GR* mRNA expression in *GR*^(+/+)^ mouse embryos was significantly greater (*P* = .004) compared to *GR*^(+/−)^ and *GR*^(−/−)^ mice. Importantly, *GR* mRNA expression for each genotype was significantly different from each other in the pituitary (*P* = .0001), with levels for *GR*^(+/+)^ embryos being the greatest, *GR*^(+/−)^ embryos being intermediate, and *GR*^(−/−)^ embryos being the lowest. These results indicate that *GR* mRNA levels were suppressed in the anterior pituitary, heart, and liver of cre-positive *GR*^(−/−)^ mice.

### Relative Pituitary Hormone mRNA Expression in *GR*(−/−) Mice Embryos

**Figure 3.**
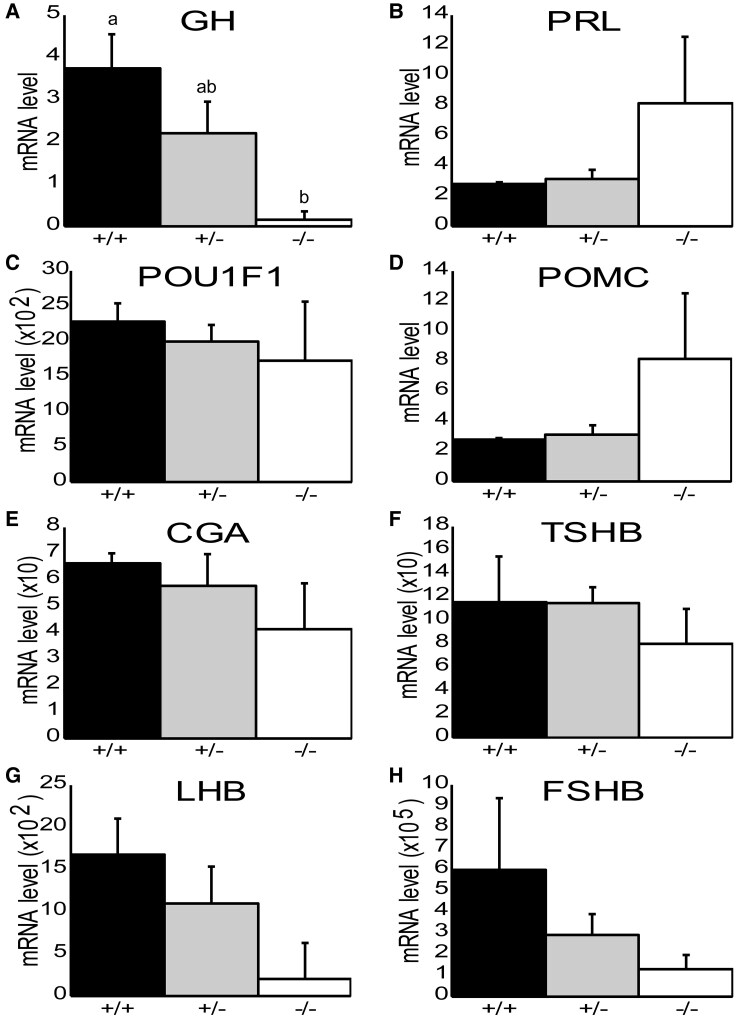
Relative *Gh*, *Prl*, *Pou1f1*, *Pomc*, *Cga*, *Tshβ*, *Lhβ*, and *Fshβ* mRNA expression in the pituitary of e17.5/e18.5 mice. Pituitaries were dissected from e17.5/e18.5 mice (*n* = 11). DNA was collected from tail snips to genotype each embryo as *GR*^(+/+)^ (*n* = 3), *GR*^(+/−)^ (*n* = 5), or *GR*^(−/−)^ (*n* = 3). Total RNA was isolated from all tissues for RT-qPCR. Data are shown as means ± SE. One-way ANOVAs were conducted for each gene, followed by Tukey's tests for post hoc analysis. Significant differences between genotypes for each gene are shown using different letters. (A) *Gh* mRNA expression was significantly (*P* = .01) reduced in *GR*^(−/−)^ embryos compared to wild-type embryos. *Gh* mRNA expression in *GR*^(+/−)^ embryos was intermediate, not significantly different from either *GR*^(+/+)^ or *GR*^(−/−)^ embryos. (B) *Prl* mRNA expression was not significantly different between any genotype. (C) *Pou1f1* (*Pit1*) mRNA expression (×10^2^ y-axis) was not significantly different between any genotype. (D) *Pomc* mRNA expression was not significantly different between any genotype. (E) *Cga* mRNA expression (×10 y-axis) was not significantly different between any genotype. (F) *Tshβ* mRNA expression (×10 y-axis) was not significantly different between any genotype. (G) *Lhβ* mRNA expression (×10^2^ y-axis) was not significantly different between any genotype. (H) *Fshβ* mRNA expression (×10^5^ y-axis) was not significantly different between any genotype.

Figure 3 presents relative mRNA expression of multiple pituitary-related genes by genotype, including growth hormone (*GH*), prolactin (*PRL*), pituitary-specific transcription factor 1 (*Pit1*), proopiomelanocortin (*POMC*), alpha-glycoprotein subunit (*αGSU*), thyroid-stimulating hormone beta (*TSHβ*), luteinizing hormone beta (*LHβ*), and follicle-stimulating hormone beta (*FSHβ*). One-way ANOVAs were conducted for each gene, followed by Tukey's tests for post hoc analysis. Significant differences between genotypes for each tissue are shown using different letters. Expression of *GH* mRNA was significantly reduced (*P* = .01) in *GR*^(−/−)^ mice (*n* = 3) compared to wild-type (*n* = 3) mice. Interestingly, levels of *GH* mRNA in heterozygous mice (*n* = 5) were intermediate, not significantly different from either wild-type or floxed mice. *PRL* (*P* = .53), *Pit1* (*P* = .65), *POMC* (*P* = .12), *αGSU* (*P* = .38), *TSHβ* (*P* = .46), *LHβ* (*P* = .17), and *FSHβ* (*P* = .31) were all not significantly different among genotypes. Although levels of mRNA for *POMC*, *LHβ*, and *FSHβ* tended to be different between genotypes, these differences were not significant with the number of litters we analyzed. These results indicate that, of the hormone-related genes analyzed, only *GH* mRNA levels were significantly reduced in the pituitary glands of cre-positive *GR*^(−/−)^ mice.

## Discussion

The regulation of growth hormone (*GH*) mRNA expression by glucocorticoids (GCs) has been extensively studied in the past, however contradicting results have led to some discrepancies as to whether glucocorticoids negatively or positively regulate *GH* mRNA expression. Traditionally, GCs are thought to negatively regulate *GH* mRNA expression (30). For example, individuals diagnosed with Cushing disease, a state of continuous cortisol overproduction, display low levels of *GH* mRNA production, along with reduced growth, and other metabolic disorders (31). However, a growing body of evidence argues that GCs are important for somatotroph differentiation and GH production during embryonic or fetal development (3, 4, 9, 13, 26). The different effects of GCs on GH expression are most likely explained either by differences between species, in vivo vs in vitro data, developmental stage or physiological state of an animal, the concentration of GCs, and long-term vs short-term effects (32).

Since chicken embryos develop in incubated eggs rather than in utero, stages of development can be accurately timed, and embryos can be easily manipulated. Embryos can be incubated for various lengths of time after manipulation. The same cannot be said for mice or rats, which is most likely why the majority of previous literature investigating CORT effects of GH regulation and somatotroph differentiation has been conducted in vitro, using chickens as a model (3-19). Despite numerous studies indicating that glucocorticoids prematurely induce differentiation of somatotrophs, the mechanism is not thoroughly understood. Studies exploring this mechanism in chicken embryonic pituitary cells indicate that CORT leads to increased *GH* transcriptional activity, rather than increased mRNA stability (33). *GR* was demonstrated to be necessary for CORT to elicit a GH response using a *GR* antagonist (14). Ras and ERK1/2 signaling have been shown to be involved in CORT induction of *GH* during embryogenesis as well (33), and the glucocorticoid responsive region of the chicken *GH* gene has been identified (18). Thus, substantial evidence has implicated CORT and *GR* in the regulation of GH expression and somatotroph differentiation. However, a requirement for *GR* has not been evaluated in vivo using a knockout animal model. Investigating the effects of a pituitary-targeted *GR*^(−/−)^ knockout in a chicken model for *GH* mRNA expression would be impractical because of the difficulties in creating transgenic chickens. Therefore, we generated a pituitary-targeted *GR* knockout (*GR*^(−/−)^) mouse model to investigate the role of *GR* in embryonic *GH* expression in a mammalian model. We elected to use the *αGSU*-cre mice, because in this transgenic model the Cre recombinase enzyme is expressed in all pituitary cells by e12.5, prior to somatotroph differentiation (23). We reasoned that combining this model with the floxed *GR* model (24) would eliminate GR from all pituitary cells, including the somatotroph precursor cells.

Mice positive for αGSU-cre and heterozygous (*GR*^(+/−)^) for the floxed *GR* allele were bred, and mouse embryos (e17.5/e18.5) were dissected out of dams to isolate brain, pituitary, heart, liver, and muscle tissue. In the pituitary, both *GR* and *GH* mRNA expression were significantly decreased in the cre-positive, homozygous *GR* knockout (*GR*^(−/−)^) mouse embryos. We speculate that both copies of the *GR* gene (*Nr3c1*) must be actively transcribed in wild-type (*GR*^(+/+)^) mice pituitaries because cre-positive *GR*^(+/−)^ mice, which possess only one copy of *Nr3c1* due to cre expression knocking out the other copy, displayed intermediate levels of *GH* mRNA and *GR* mRNA in the pituitary of *GR*^(+/−)^ mice relative to *GR*^(+/+)^ and *GR*^(−/−)^ mice. These results demonstrate an essential role for *GR* in the pituitary for normal *GH* mRNA ontogeny and somatotroph differentiation in mice.

We also analyzed several other pituitary-related genes to determine whether knocking out *GR* had any additional effects on their expression. The POU homeodomain protein PIT1, a pituitary-specific transcription factor, is expressed in mice by e15.5 (34). Pit1 is believed to be essential for proper differentiation of somatotrophs, thyrotrophs, and lactotrophs since they are not observed in *Pit1* mutant mice. Additionally, *GH*, *TSHβ*, and prolactin (*PRL*) mRNA are not detected in *Pit1* mutant mice (34). When we analyzed *Pit1* mRNA expression, we found no significant differences between genotypes. These results provide evidence that *GR* knockout does not alter *Pit1* mRNA expression. Therefore, the reduction of *GH* mRNA levels observed in *GR*^(−/−)^ mice is likely due to a lack of *GR* and not due to effects on PIT1. When we analyzed the expression of *αGSU, FSHβ, LHβ,* and *TSHβ*, we found no significant differences between genotypes in *αGSU* or *TSHβ* mRNA expression. Interestingly, *FSHβ and LHβ* mRNA appeared to be reduced in *GR*^(−/−)^ embryos; however, with our sample size, these differences in gene expression were not significantly different between genotypes. These results indicate that *GR* does not regulate the expression of *αGSU, FSHβ, LHβ,* or *TSHβ* mRNA in the mouse during embryonic development. We also analyzed the expression of *POMC* and *PRL* mRNA in the pituitary. We found no significant differences in gene expression for any of these genes, across all genotypes. Noteworthy, *POMC* mRNA expression appeared to be upregulated in *GR*^(−/−)^ mice, although this was not statistically significant. *POMC* mRNA expression may have been upregulated if the negative feedback loop between glucocorticoids and the hypothalamic-pituitary-adrenal (HPA) axis was interrupted. Expression of *GR* mRNA in brain tissue did not show any significant differences between any genotypes. Our findings indicate that pituitary-targeted knockout of *GR* suppressed mRNA levels in the pituitary for only *GH* but not the other pituitary hormone genes.

Based on the findings in our transgenic model, we conclude that *GR* expression in the pituitary is required during embryogenesis for normal *GH* ontogeny in mice. Additionally, other pituitary-related genes analyzed in this study, including *Pit1*, *PRL*, *POMC*, *αGSU*, *TSHβ*, *FSHβ*, and *LHβ*, were not significantly different between genotypes. Thus, the effect of pituitary-targeted knockout of *GR* was specific to *GH*, suppressing normal *GH* expression during embryonic development. To the best of our knowledge, this is the first in vivo investigation to demonstrate that *GR* expression in the pituitary is required during embryogenesis for normal *GH* mRNA expression and somatotroph differentiation in mouse embryos.

From the onset of this study, we discovered that the *GR* gene was not following Mendelian genetics in the mouse pups after birth. During embryogenesis, however, the heredity pattern of the *GR* gene indicated that Mendelian genetics were being followed. At some point between embryonic day 18.5 and birth all of the cre-positive *GR*^(−/−)^ pups died. These results indicate that homozygous knockout of *GR* in some tissue is a neonatal lethal phenotype, however the cause of death is not clear. Previous studies utilizing the *αGSU* promoter in mice to drive *cre* expression have reported *cre* expression on e9.5 in tissues outside of the pituitary, including in muscle and cardiac tissue, with little or no expression in gonads, adrenal glands, kidneys, brain, or ventromedial hypothalamus (23). While the only tissue in which we detected *cre* expression was the pituitary on e17.5/e18.5, *GR* mRNA was also significantly suppressed in the heart of *GR*^(−/−)^ embryos and in the liver of *GR*^(+/−)^ and *GR*^(−/−)^ embryos compared to *GR*^(+/+)^ embryos. A previous study demonstrated that knocking out *GR* in cardiomyocytes led to cardiac hypertrophy and death. However, these mice appeared normal at birth and did not show symptoms of heart failure until around 5 months of age (35). A different study that conducted in situ hybridization in adult mouse tissues, using β-galactosidase as a reporter gene for *αGSU* expression, found no significant expression in the liver (36). It should be noted that if *αGSU* is expressed at any period in a cell, cre recombinase will be synthesized and will excise the floxed region of the *GR* gene. Therefore, it is theoretically possible that *cre* was expressed in cardiac and liver tissue early in development through the *αGSU* promoter, and the floxed region of the *GR* gene was removed from the genome of these cells, but we did not detect *cre* on e17.5/e18.5 because *αGSU* is no longer expressed in the tissue at that point. While *αGSU* is traditionally considered to be a pituitary-specific gene, these results, as well as previous reports, indicate the ontogeny and tissue distribution of *αGSU* expression may not be completely understood. *GR* has been shown to be necessary for lung development in mice as well (37). Since we did not collect lung tissue from our embryo samples, we can only speculate if improper lung development contributed to neonatal lethality. Although we do not know the basis for the neonatal phenotype in our cre-positive *GR*^(−/−)^ pups, our findings clearly demonstrate that pituitary *GH* and *GR* mRNA expression were significantly decreased in cre-positive *GR*^(−/−)^ embryos.

In conclusion, our findings indicate that pituitary-targeted knockout of *GR* suppresses the normal ontogeny of *GH* mRNA expression during mouse development. This finding, in combination with the substantial evidence that CORT can induce premature expression of GH during embryonic development, indicates an essential role for CORT and *GR* in normal somatotroph differentiation.

## Data Availability

All datasets generated during and/or analyzed during the current study are not publicly available but are available from the corresponding author on reasonable request.

## Abbreviations

CORT: corticosterone
e#: embryonic day #
FSH: follicle-stimulating hormone
GC: glucocorticoid
GH: growth hormone
GR: glucocorticoid receptor
LH: luteinizing hormone
PCR: polymerase chain reaction
PRL: prolactin
RT-qPCR: reverse transcription–quantitative polymerase chain reaction
TSH: thyrotropin (thyroid-stimulating hormone)
